# Beliefs, Knowledge, Implementation, and Integration of Evidence-Based Practice Among Primary Health Care Providers: Protocol for a Scoping Review

**DOI:** 10.2196/resprot.7727

**Published:** 2017-08-01

**Authors:** Filipa Pereira, Mireille Salvi, Henk Verloo

**Affiliations:** ^1^ School of Health Sciences, HES-SO Valais/Wallis Department of Nursing Sciences University of Applied Sciences and Arts of Western Switzerland Sion Switzerland

**Keywords:** evidence-based practice, primary healthcare, primary healthcare providers, beliefs, knowledge, implementation

## Abstract

**Background:**

The adoption of evidence-based practice (EBP) is promoted because it is widely recognized for improving the quality and safety of health care for patients, and reducing avoidable costs. Providers of primary care face numerous challenges to ensuring the effectiveness of their daily practices. Primary health care is defined as: the entry level into a health care services system, providing a first point of contact for all new needs and problems; patient-focused (not disease-oriented) care over time; care for all but the most uncommon or unusual conditions; and coordination or integration of care, regardless of where or by whom that care is delivered. Primary health care is the principal means by which to approach the main goal of any health care services system: optimization of health status.

**Objective:**

This review aims to scope publications examining beliefs, knowledge, implementation, and integration of EBPs among primary health care providers (HCPs).

**Methods:**

We will conduct a systematic scoping review of published articles in the following electronic databases, from their start dates until March 31, 2017: Medical Literature Analysis and Retrieval System Online (MEDLINE) via PubMed (from 1946), Embase (from 1947), Cumulative Index to Nursing and Allied Health Literature (CINAHL; from 1937), the Cochrane Central Register of Controlled Trials (CENTRAL; from 1992), PsycINFO (from 1806), Web of Science (from 1900), Joanna Briggs Institute (JBI) database (from 1998), Database of Abstracts of Reviews of Effects (DARE; from 1996), Trip medical database (from 1997), and relevant professional scientific journals (from their start dates). We will use the predefined search terms of, “evidence-based practice” and, “primary health care” combined with other terms, such as, “beliefs”, “knowledge”, “implementation”, and “integration”. We will also conduct a hand search of the bibliographies of all relevant articles and a search for unpublished studies using Google Scholar, ProQuest, Mednar, and WorldCat. We will consider publications in English, French, Spanish, and Portuguese.

**Results:**

The electronic database searches were completed in April 2017. Retrieved articles are currently being screened, and the entire study is expected to be completed by November 2017.

**Conclusions:**

This systematic scoping review will provide a greater understanding of the beliefs, knowledge, implementation, and integration of EBPs among primary HCPs. The findings will inform clinical practice and help to draw a global picture of the EBP research topics that are relevant to primary care providers.

## Introduction

Evidence-based practice (EBP) is an emerging, breakthrough approach among health care providers (HCPs) [1,2]. EBP has its origins in evidence-based medicine and is defined as, “the conscientious and judicious use of current best evidence in making decisions about the care of individual patients” [3]. HCPs are expected to use EBPs as a standard approach to daily practice [4,5], thereby integrating research, patient preferences, clinical expertise, and innovative technologies [6-8]. However, the implementation of EBPs remains a controversial process [9,10], and not all HCPs are convinced that it improves the quality of care [11,12]. Implementing EBPs is challenging, especially in primary health care settings [13,14].

Primary health care is defined as: the entry level into a health care services system, providing a first point of contact for all new needs and problems; patient-focused (not disease-oriented) care over time; care for all but the most uncommon or unusual conditions; and coordination or integration of care, regardless of where or by whom that care is delivered. Primary health care is the primary means by which to approach the main goal of any health care services system: optimization of health status [15]. Health care provided by primary HCPs includes health promotion, prevention and diagnosis, detection, intervention, treatment, and case and care management [16,17]. Furthermore, primary HCPs provide first-line health care services to home-dwelling adult patients and long-term nursing home patients. Nevertheless, in some acute health situations, home-dwelling individuals will need to be referred to medical specialists or acute hospital services for additional health care advice [15,17]. Primary HCPs play a crucial decision-making role, which strengthens communication and collaboration between community HCPs and specialized HCPs to provide the best available overall health care to community-dwelling individuals [14]. Primary HCPs include general practitioners, community health care nurses and nurse practitioners, midwives and allied health care professionals (occupational therapists, physical therapists, speech and language therapists, podiatrists, dieticians, psychologists, social workers, and radiological and medical imaging technologists), pharmacists, and dentists [18].

Although every HCP is generally considered accountable for providing the best available evidence-based health care [19,20], recent research has concluded that only a small percentage of HCPs consistently do so [21-25]. The implementation rate of EBPs among HCPs in hospital institutions has been largely documented [26,27] and multiple barriers have been reported [19,22,28,29]. These barriers include time constraints, a lack of personal motivation and negative attitudes, professional resistance to research, and inadequate knowledge of (and skills needed for) EBPs among clinicians [30-32]. Additionally, several authors have documented administrative and organizational problems in the workplace, a lack of mentors for EBPs, inadequate resources at the point of care, a gap between theory and practice, a lack of any meaningful transition between training courses on EBP and the clinical reality, and an absence or lack of basic education on the subject [21,25,33]. Finally, different authors have highlighted that HCPs’ beliefs about EBPs are associated with their capacity to implement such practices [22,34,35].

Over the last two decades, the use of EBPs in health care has been documented in exploratory and observational studies in different settings. However, there have been no wide-ranging overviews or comparisons examining the beliefs, knowledge, implementation, and integration of EBPs in primary health care settings, or among primary HCPs [36,37].

This scoping review aims to explore the different studies examining the beliefs, knowledge, implementation, and integration of EBPs in primary health care settings and among primary care HCPs. The following research questions will be explored:

1. What is the extent of the research exploring beliefs, knowledge, implementation, and integration of EBPs among primary HCPs?
2. What is the nature of the research exploring beliefs, knowledge, implementation, and integration of EBPs among primary HCPs?
3. How do the extent and nature of the research exploring beliefs, knowledge, implementation, and integration of EBPs vary across primary HCPs?

## Methods

We will use the scoping review methodological framework conceived by Arksey and O'Malley [38], with the refinements described by Levac et al [39] and Colquhoun et al [40], and consider the recommendations of the Preferred Reporting Items for Systematic Reviews and Meta-Analyses Protocols (PRISMA-P) statement [41].

This multistage model involves: (1) identifying the research questions (listed above); (2) identifying relevant studies (search methods used); (3) selecting studies; (4) charting the data; (5) collating, summarizing, and reporting the results; and, if necessary, (6) consulting with key stakeholders. The refinements to the original framework include: establishing clear research questions, purposes, and expected outcomes of the scoping review; assembling a team with content and methodological expertise; searching the literature using an iterative process involving inclusion and exclusion criteria; using at least two reviewers to independently review abstracts and full-text papers, with a consensus procedure in cases of disagreement; developing a data extraction form onto which two researchers can upload the data independently; conducting a quality assessment of included papers; and performing an analysis that includes a descriptive quantitative summary of papers, as well as a qualitative thematic analysis.

### Eligibility Criteria

Searches in scoping reviews are recommended to be as comprehensive as possible, in order to identify every possible study that is relevant to the field [39,42]. Original studies eligible for inclusion encompass descriptive studies, cohort studies, interventional studies, qualitative studies, mixed-design studies, and reports studying EBPs among primary HCPs. Studies should provide a measure or level of: (1) belief, (2) knowledge, (3) implementation, or (4) integration of EBPs, as defined by the authors.

Studies should be conducted among primary HCPs in primary health care settings, and among HCPs carrying out parallel activities in primary, secondary, and tertiary health care settings. Studies should also include regional and national surveys among general practitioners, community health care nurses and nurse practitioners, midwives, occupational therapists, physical therapists, speech and language therapists, podiatrists, dieticians, psychologists, social workers, radiological and medical imaging technologists, dentists, and pharmacists. Grey literature containing information on beliefs, knowledge, implementation, and integration of EBPs is eligible. Studies including expert opinions and editorials will be excluded from the original set of studies collected.

### Information Sources and Search Strategy

We will conduct a systematic scoping search of published articles in the following electronic databases, from their start dates until March 31, 2017: Medical Literature Analysis and Retrieval System Online (MEDLINE) via PubMed (from 1946), Embase (from 1947), Cumulative Index to Nursing and Allied Health Literature (CINAHL; from 1937), the Cochrane Central Register of Controlled Trials (CENTRAL; from 1992), PsycINFO (from 1806), Web of Science (from 1900), Joanna Briggs Institute (JBI) database (from 1998), Database of Abstracts of Reviews of Effects (DARE; from 1996), Trip medical database (from 1997) and relevant professional scientific journals for the primary HCPs mentioned (from their start dates). We will use predefined search terms and Medical Subject Headings (MeSH) terms such as, “evidence-based practice”, “evidence-based nursing”, “evidence-based approach”, “best practices”, “beliefs”, “knowledge”, “implementation”, “integration”, and “health personnel”. The “health personnel” MeSH term’s tree will be expanded using the MeSH terms and keywords “health care” and “allied health care professionals”. In addition to searching electronic databases, we will conduct a hand search of the bibliographies of all relevant articles and a search of unpublished studies using Google Scholar, ProQuest, Mednar, the WorldCat and Health Technology Assessment (Canadian Search Interface) databases, and OpenGrey. We will consider publications in English, French, Spanish, and Portuguese. If studies are identified in languages other than those mastered by the research team, we will contact their authors to complete the data extraction and quality assessment form. A draft MEDLINE search strategy is included in Multimedia Appendix 1. When the MEDLINE strategy has been finalized, subject headings and syntax will be adapted for the other databases. The search strategy’s results will be reported following the PRISMA statement guidelines (Multimedia Appendix 2) [43].

### Bibliographic Management

Review Manager software version 5.3 and Endnote 8.0 will be used for collecting and analyzing the bibliographic references retrieved using the search strategy [44].

### Article Selection Process

We will include studies by using a two-step process with two independent reviewers for each step. First, each abstract initially selected will be evaluated. Then, each potentially relevant full article will be retrieved for consideration of inclusion. Second, data will be selected and extracted. In cases of a disagreement between the two reviewers, the other team members will contribute to the decision. Disagreements will be resolved through discussion among the team members or, if needed, a consensus will be reached following a further discussion with the authors. Article selection will be based on the methodological framework for scoping studies recommended by Arksey and O'Malley and Levac et al [38,39] and the systematic review by Leung et al [45].

### Data Collection Process

We will use standardized data collection forms (Microsoft Excel sheets for the data on the studies and interventions, as well as a quality assessment of the studies included) developed by the research team. Data extraction will be conducted independently by two separate reviewers using a specially designed, standardized data extraction form based on a relevant, previously published extraction form [46]. Discrepancies will be resolved through discussion and consultation with the co-authors.

### Data Items

The following information will be extracted from each included study and put into an appropriate usable form: (1) study author, year of publication, and country in which the study was conducted; (2) study characteristics (including research questions, study setting and design, instruments used, duration of follow-up, and sample size); (3) participants’ characteristics (including sex, age, professional activity and experience, level of education, and setting); and (4) types of outcome measures [47].

### Outcomes and Prioritization

The primary outcomes will be the nature, number, and comparison of studies examining beliefs, knowledge, implementation, and/or integration of EBPs among primary HCPs. These outcomes will allow us to generate an overview of the existing implementation and integration of EBPs in primary health care.

We hypothesize that significant differences will exist in the designs and instruments of EBPs, both in different primary health care settings and among different primary HCPs. Secondary outcomes will focus on: the beliefs, knowledge, implementation, and integration measures/levels of EBPs; the theoretical frameworks and instruments used for assessment; and the level of evidence provided in the included studies [48]. Overall, the outcomes of this scoping review will provide useful suggestions and recommendations for identifying possible gaps in the research [38].

### Data Synthesis

We will summarize the results using a descriptive (noninterpretive) narrative synthesis [38,39,42] and content analysis [49]. All data on the beliefs, knowledge, implementation, and integration measures/levels for EBPs will be summarized in a table. By summarizing and critically appraising all studies, we will be able to identify gaps in the current evidence and avenues for future research.

### Confidence in Cumulative Evidence and Risk of Bias

As recommended by Daudt et al [42], we will assess the quality of the eligible observational and mixed-method studies by using the appropriate recommended tools: Grading of Recommendations Assessment, Development, and Evaluation; and the Mixed-Methods Appraisal Tool [47,50]. Qualitative studies will be assessed using the Letts Qualitative Review Form [51]. The risk of bias in the retrieved studies will be assessed using the Cochrane Collaboration tool [44]. We will not, however, exclude studies based on a quality assessment, because we wish to provide a comprehensive overview of the available evidence and its extent. The quality of evidence will be assessed using the following items: risk of bias, inconsistency, imprecision, indirectness, publication bias, and the confidence effect [44]. Using these tools, each of the following domains will be rated as: (1) very low quality, (2) low quality, (3) moderate quality, or (4) high quality [52]. Any disagreements regarding the quality assessment will be resolved through discussion among the team members.

### Ethics and Dissemination

All data in this project will be gathered through searches of literature databases, and recommendations and guidelines available online. No information on individuals will be collected within the framework of this project, thus approval from a research ethics committee is not required.

## Results

The electronic database searches were completed in July 2017. Retrieved articles are currently being screened and the entire study is expected to be completed by November 2017.

## Discussion

Providing the best available, safe, high-quality health care is the gold standard objective in all health care settings. To the best of our knowledge, there exists no global overview of the beliefs, knowledge, implementation, and integration of EBPs among primary HCPs and institutions. This documentary research project will provide a picture of the state of the art of research in this domain, and reveal to what extent EBPs are implemented and integrated. This review will, therefore, provide valuable information to practitioners, policy makers, and other stakeholders.

### Strengths and Limitations

This systematic scoping research protocol’s strengths are: (1) obtaining a broad overview of the studies dealing with beliefs, knowledge, implementation, and integration of EBPs among primary HCPs; (2) the use of an appropriate search strategy designed in collaboration with two specialist librarians who are experienced in conducting such reviews; and (3) the inclusion criteria, which impose no restrictions on time period or geographic location.

Nevertheless, this protocol does include some limitations which may introduce bias: (1) the exclusion of articles written in languages other than English, French, Spanish, or Portuguese; and (2) the personal judgements of the reviewers’ study assessments.

## Appendix

Multimedia Appendix 1 Draft MEDLINE - PubMed search.

Multimedia Appendix 2 Flow diagram based on the PRISMA-p guidelines.

## Abbreviations

CENTRAL: Cochrane Central Register of Controlled Trials
CINAHL: Cumulative Index to Nursing and Allied Health Literature
DARE: Database of Abstracts of Reviews of Effects
EBP: evidence-based practice
HCP: health care provider
JBI: Joanna Briggs Institute
MEDLINE: Medical Literature Analysis and Retrieval System Online
MeSH: Medical Subject Headings
PRISMA-P: Preferred Reporting Items for Systematic Reviews and Meta-Analyses Protocols
