# Supplementary material for: Beliefs, Knowledge, Implementation, and Integration of Evidence-Based Practice Among Primary Health Care Providers: Protocol for a Scoping Review
Source: JMIR Res Protoc. 2017 Aug 1;6(8):e148. doi: 10.2196/resprot.7727 (PMC5558043; doi:10.2196/resprot.7727)
Supplement: Multimedia Appendix 1 [file resprot_v6i8e148_app1.pdf]

Multimedia Appendix 1

Draft MEDLINE - PubMed search

The following equation is an example of the search strategy without language restrictions for published and unpublished papers:

- MEDLINE PubMed (1946 to 2016)

S1 “Evidence-based Practice” [Mesh] OR "EBP" [All fields] OR "evidence-based pratice" [Mesh] OR "evidence-based pratice" [tiab] OR evidence based* [tiab] OR "evidence-based pratice" [All fields]

S2 “Health Personnel” [Mesh] OR “Physicians” [Mesh] OR “physicians*” [tiab] OR "Nurses" [Mesh] OR “Nurses*” [tiab] OR “Nurse Midwives” [Mesh] OR “Midwives*” [tiab] OR "allied health occupations" [Mesh] OR "allied healthcare professionals*" [tiab] OR "Physical therapist" [Mesh] OR "Occupational therapist" [Mesh] OR “Midwives” [Mesh] OR “allied healthcare professionals*” [tiab] OR "Occupational Therapy" [Mesh] OR "Occupational Therapists" [Mesh] OR "occupational therapists*" [tiab] OR

"radiological technologists*" [tiab] OR "radiologists" [tiab] OR "Radiologists" [Mesh] OR "imaging technologists*" [tiab] OR "radiologists*" [tiab] OR "community nurses*" [tiab]) OR "community health nursing" [Mesh]) OR (("community healthcare" [tiab] AND "nurses*" [tiab] OR "Nurse practitioners*" [tiab] OR "Nurse Practitioners" [Mesh] OR "social workers*" [tiab] OR "social workers" [Mesh] OR "psychologists*" [tiab] OR "psychology" [Mesh]

S3 "Knowledge" [Mesh] OR "Belief*" [Mesh] OR "Implementation" [Mesh] OR EBP steps* [tiab] “Knowledge” [Mesh] OR “health knowledge, attitudes, practice” [Mesh] OR “Knowledge” [tiab] OR “Belief” [tiab] OR “beliefs” [tiab]) AND “culture” [Mesh] NOT “religion” [Mesh] OR "implement" [All Fields] OR "implementation" [All Fileds] OR "implementing" [All Fileds]

S4 EBP frame Surveys and Questionnaires* [Mesh] OR “Question*” [tiab] OR “tool*” [tiab] OR “model*” [tiab] OR “framework*” [tiab] OR “Guidelines as Topic” [Mesh] OR “guideline*” [tiab] OR “best practice*” [tiab]

S5 “Assessment*” [tiab] OR “measurement*” [tiab] OR “application*” [tiab] OR “integration*” [tiab]

S6 “Primary health care” [Mesh] OR “primary care” [tiab] "primary health care" [tiab] OR “Primary Nursing” [Mesh] OR “Primary Nursing” [tiab]

S7 S1 AND S2 AND S3 AND S4 AND S5 AND S6
