# Supplementary figures and images for: Beliefs, Knowledge, Implementation, and Integration of Evidence-Based Practice Among Primary Health Care Providers: Protocol for a Scoping Review
Source: JMIR Res Protoc. 2017 Aug 1;6(8):e148. doi: 10.2196/resprot.7727 (PMC5558043; doi:10.2196/resprot.7727)

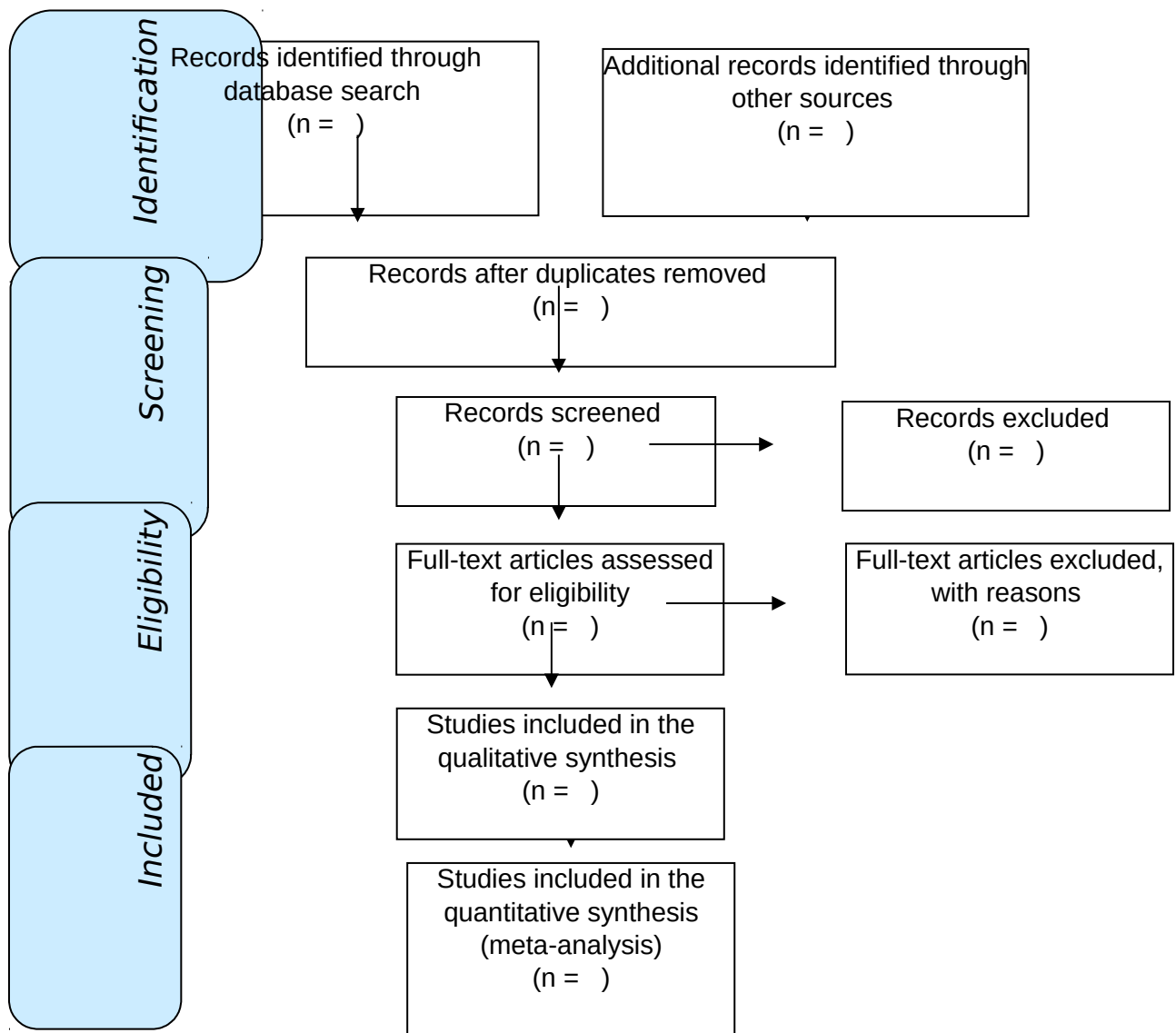

Figure 1. Flow diagram based on the PRISMA-p guidelines [41]

Supplement: Multimedia Appendix 2 [file resprot_v6i8e148_app2.pdf]
